# An Improved Locally Weighted PLS Based on Particle Swarm Optimization for Industrial Soft Sensor Modeling

**DOI:** 10.3390/s19194099

**Published:** 2019-09-22

**Authors:** Minglun Ren, Yueli Song, Wei Chu

**Affiliations:** 1 School of Management, Hefei University of Technology, Hefei 230009, China; chunews@163.com; 2 Key Laboratory of Process Optimization and Intelligent Decision-making, Ministry of Education, Hefei 230009, China

**Keywords:** locally weighted PLS, particle swarm optimization, just-in-time learning, bandwidth parameter, soft sensor

## Abstract

In industrial production, soft sensors play very important roles in ensuring product quality and production safety. Traditionally, global modeling methods, which use historical data to construct models offline, are often used to develop soft sensors. However, because of various complex and unknown changes in industrial production processes, the performance of global models deteriorates over time, and frequent model maintenance is difficult. In this study, locally weighted partial least squares (LWPLS) is adopted as a just-in-time learning method for industrial soft sensor modeling. In LWPLS, the bandwidth parameter h has an important impact on the performance of the algorithm, since it decides the range of the neighborhood and affects how the weight changes. Therefore, we propose a two-phase bandwidth optimization strategy that combines particle swarm optimization (PSO) and LWPLS. A numerical simulation example and an industrial application case were studied to estimate the performance of the proposed PSO–LWPLS method. The results show that, compared to the traditional global methods and the LWPLS with a fixed bandwidth, the proposed PSO–LWPLS can achieve a better prediction performance. The results also prove that the proposed method has apparent advantages over other methods in the case of data density changes.

## 1. Introduction

In industrial production, in order to ensure product quality or production safety, it is necessary to monitor the key process parameters or quality indicators in real time. However, online measurements are not always available, because the experimental analysis may be very time consuming, or analytical instruments may exceed acceptability. In these cases, soft sensors have been proposed and proved to be very practical, which are usually predictive models or systems built by using abundant data collected from industrial production. In recent years, soft sensors have become more and more popular in quality prediction and other applications related to process control [1,2].

According to a survey conducted by Japanese scholars, more than 90% of soft sensor models used in the Japanese chemical industry are based on linear modeling methods, including multiple linear regression (MLR), partial least squares (PLS), principal component analysis (PCA), and so on [3]. In order to adapt to the nonlinearity of some industrial processes, several nonlinear modeling methods are also used, such as artificial neural network (ANN) [4,5], support vector machine (SVM), [6,7], nonlinear PLS [8], and so on. Despite the above methods being popular, it is still not easy to continually keep the soft sensors in good performance. In order to build a good soft sensor, complex process changes have to be considered; otherwise, the accuracy of the soft sensor may decrease after a period of time. To cope with this problem, some adaptive methods, such as moving window techniques [9] and recursive adaptation techniques [10,11], have been proposed. These methods can gradually adapt to new process states, but they cannot deal with abrupt changes such as catalyst failure and raw material changes [12,13].

In such situations, the just-in-time learning (JITL) method has been recently introduced to develop soft sensors, and it has successfully been used to control some industrial processes [14,15,16,17,18]. In the JITL framework, there is no model that needs to be constructed offline in advance. When a prediction is required, the weight of each sample in the historical dataset is first calculated according to its similarity to the query sample; then, a local PLS model is constructed. After answering the query, the model is discarded. This means that every time a query arrives, the JITL-based method builds a new local model [18]. Therefore, the JITL-based method can better cope with process changes, compared to global methods, and local model reconstruction also enables it to cope well with nonlinear processes [17]. 

As one kind of JITL method, locally weighted PLS (LWPLS) can cope well with nonlinearity, collinearity, and process changes by combining the properties of PLS and locally weighted regression (LWR) [19]. Therefore, it is very popular in various industrial applications. In LWPLS, several parts closely related to its performance need to be well selected, such as the bandwidth parameter, distance function, weight function, and so on. This paper focuses on the bandwidth parameter *h*, which can be crucial to LWPLS, since it determines the range of the neighborhood and the shape of weight changes when a local PLS model needs to be constructed [20,21]. If the bandwidth *h* is too small, the problem of overfitting will occur, and the reliability of the LWPLS model will also be affected. On the other hand, if it is too large, satisfactory accuracy will not be achieved [21,22]. Therefore, a scheme is required to decide the optimal bandwidth parameter.

Several typical methods have been used to determine this parameter. One method is to use a fixed bandwidth. Although this method is simple, it cannot achieve a satisfactory performance because the data density in the whole data set is likely to be very different. Another method is the k-nearest neighbor (K-NN) bandwidth method. In this method, the distance between the query point and each sample is first calculated and arranged in ascending order; then, the bandwidth is set to be the K-th distance value [23]. Compared to the fixed bandwidth selection, the K-NN method performs better in various applications. However, it is not easy to determine an optimal K value for each query [20,21,23]. The third method is the automatic bandwidth design method, in which the optimal bandwidth is determined by trial and error based on certain criteria, such as the minimum mean square error (MMSE) criterion or Akaike’s information criterion (AIC) [22]. This method lacks the idea of optimization, and the accuracy needs to be further improved. In addition, some intelligent algorithms, such as the genetic algorithm (GA) [21,24] and particle swarm optimization (PSO) [22], have been used to optimize the bandwidth. However, in these methods, bandwidth optimization, using the intelligent algorithm, is done online, which greatly affects the query response speed of the model.

To improve the model’s accuracy, while at the same time ensuring a rapid query response, a two-phase bandwidth optimization strategy, including a training phase and a prediction phase, is proposed by combining PSO and LWPLS. The proposed method is referred to as PSO-LWPLS, which integrates the basic idea of point-based local bandwidth selection [20] and the K-NN method. Both offline and online operation schemes are designed to obtain the optimal bandwidth for each query. However, this is a single parameter optimization problem: why is PSO used instead of traditional one-dimensional search methods such as the Newton method, or the golden section search method (GSSM)? The objective of bandwidth optimization is to minimize the prediction error, so the relationship between the objective function and the bandwidth parameter is analyzed. The analysis results show that this relationship is complex and cannot be expressed by explicit mathematical formulas. In addition, when the density distribution of sample data is different, multiple minimum values could occur in the relationship curves between the objective function and the bandwidth value. The above two factors make the traditional one-dimensional search methods not applicable. This will be explained in detail in Section 3.2.

The rest of this paper is arranged as follows. Section 2 briefly describes the basic LWPLS and PSO algorithms. Then, in Section 3, the proposed PSO-LWPLS modeling method is described in detail. Both numerical and industrial case studies are given in Section 4. Finally, the paper is summarized in Section 5.

## 2. Preliminaries

### 2.1. Locally Weighted PLS

LWPLS is extended from basic PLS according to the JITL framework [25]. When an output estimation is requested, LWPLS uses PLS to construct a local model. Compared to conventional LWR, which uses multiple linear regression for local modeling, LWPLS is preferable because of its robustness and advantages in dealing with collinearity. The basic algorithm of LWPLS is explained below [26].

Let ***X*** (*N* × *p* matrix) and ***Y*** (*N* × *m* matrix) be input and output variables, which constitute a historical dataset. *N* represents the total sample number; *p* and *m* represent the input and output variable numbers, respectively. The *n*th sample is denoted by:

(1) $$X_{n}=[x_{n1},x_{n2},\cdots ,x_{np}]$$

(2) $$Y_{n}=[y_{n1},y_{n2},\cdots ,y_{nm}]$$

To obtain the predicted value of a query ***X***_q_ (1 × *p* vector), first, the weight corresponding to each sample ***X***_n_ (n = 1, 2, …, *N*) is calculated according to the following equations:

(3) $$\omega n=\exp(-\frac{dn}{h\times \sigma_{d}})$$

(4) $$d_{n}=\sqrt{(X_{n}-X_{q}){\left(X_{n}-X_{q}\right)}^{T}}$$

(5) $$d={\left[d_{1},d_{2},\cdots ,d_{N}\right]}^{T}$$

where $\sigma_{d}$ denotes the standard deviation of the distance vector d; and *h* is the bandwidth parameter discussed in this paper, which is also called smoothing parameter. The optimal selection of parameter *h* will be given in detail in the third section. The locally weighted PLS model is built according to the weight diagonal *N* × *N* matrix **Ω** given as follows:

(6) $$\Omega =diag[\omega_{1},\omega_{2},\cdots ,\omega_{N}]$$

The details of the LWPLS algorithm are shown in Algorithm 1. To determine the latent variable number *R*, the cross-validation method is used. In step 3, the input variable ***X***, the output ***Y***, and the query ***X***_q_ are all mean-centered in advance. Steps 5–7 iteratively derive the latent variable ***t***, loading vector ***p***, and the regression coefficient vector ***q***. To get the parametric vector ***W***_r_ in step 6, the maximum eigenvalue of $X_{r}^{T}\Omega Y_{r}Y_{r}^{T}\Omega X_{r}$ is first calculated; then, its corresponding eigenvalues are assigned to ***W***_r_ [25,27]. 


**Algorithm 1: LWPLS**
1: Set *R* and *h*.2: Calculate the weight matrix **Ω**3: Calculate ***X***_0_, ***Y***_0_, and ***X***_q0_;
(7)$$X_{0}=X-1_{N}[{\bar{x}}_{1},{\bar{x}}_{2},\cdots ,{\bar{x}}_{p}]$$

(8)$$Y_{0}=Y-1_{N}[{\bar{y}}_{1},{\bar{y}}_{2},\cdots ,{\bar{y}}_{m}]$$

(9)$$X_{q0}=Xq-[{\bar{x}}_{1},{\bar{x}}_{2},\cdots ,{\bar{x}}_{p}]$$

(10)$${\bar{x}}_{i}=\frac{\sum_{n=1}^{N}\omega_{n}x_{ni}}{\sum_{n=1}^{N}\omega_{n}}$$

(11)$${\bar{y}}_{j}=\frac{\sum_{n=1}^{N}\omega_{n}y_{nj}}{\sum_{n=1}^{N}\omega_{n}}$$
4: **Set X**r= ***X***_0_, **Y**r= ***Y***_0_, ***X***_q r_= ***X***_q0_, ${\hat{Y}}_{q}=[{\bar{y}}_{1},{\bar{y}}_{2},\cdots ,{\bar{y}}_{m}]$5: **for** r = 1, to R **do**6: Derive the rth latent variables of ***X***, ***Y***, and ***X***_q_;
(12)$$t_{r}=X_{r}W_{r},t_{qr}=X_{qr}W_{r}$$
7: Derive the rth loading vector of ***X*** and the rth regression coefficient of ***Y***;
(13)$$p_{r}=X_{r}^{T}\Omega t_{r}/t_{r}^{T}\Omega t_{r},q_{r}=Y_{r}^{T}\Omega t_{r}/t_{r}^{T}\Omega t_{r}$$
8: Update ${\hat{Y}}_{q}={\hat{Y}}_{q}+t_{qr}q_{r}^{T}$9: **if** r = R10:  Output the predicted value ${\hat{Y}}_{q}$11:  **else**12:  Calculate **X**r_+1_, **Y**r_+1_, and ***X***_q r+1_.
(14)$$X_{r+1}=X_{r}-t_{r}p_{r}^{T}$$

(15)$$Y_{r+1}=Y_{r}-t_{r}q_{r}^{T}$$

(16)$$X_{qr+1}=X_{qr}-t_{qr}p_{r}^{T}$$
13:  **end if**14:  **end for**

### 2.2. PSO Algorithm

PSO is a group search algorithm that was proposed in 1995 [28], and has been effectively used for many optimization problems. In PSO, a group of individuals is randomly arranged in the search space at first. Each individual is a particle representing a hypothetical solution. Then, the individuals constantly change their positions based on information sharing in the group, and finally, all individuals converge to the optimal solution. The fitness function, which is selected according to the specific optimization problem, is also adopted in PSO to estimate the quality of the individuals’ solutions [29].

Particle velocity and position are iteratively updated by the equations below:

(17) $$V_{i}(t+1)=\omega V_{i}(t)+c_{1}r_{1}(X_{i,\mathrm{pbest}}(t)-X_{i}(t))+c_{2}r_{2}(X_{i,\mathrm{gbest}}(t)-X_{i}(t))$$

(18) $$X_{i}(t+1)=X_{i}(t)+V_{i}(t+1)$$

where *t* and *t* + 1 represent the current iteration and the next iteration, respectively. For the *i*th particle, $V_{i}$ represents its velocity, and $X_{i}$ represents its position; $r_{1}$, $r_{2}$ are two independent, random numbers with a uniform distribution in the interval 0–1; and $c_{1}$, $c_{2}$ are learning factors, and both are non-negative constants. $X_{i,pbest}$ denotes the best position of the *i*th particle found at the current moment. $X_{gbest}$ denotes the optimal position found by all particles in the swarm. w is the inertial weight, which is an important parameter of PSO because it can balance global and local search capabilities. 

A linearly decreasing inertial weight is adopted in this paper, which is calculated by the following equation [30]:

(19) $$w^{t}=w_{\min}+(w_{\max}-w_{\min})*(t_{\max}-t)/t_{\max}$$

where *t*_max_ denotes the maximum number of iterations, *t* denotes the current iteration, and values of $w_{\min}$ and $w_{\max}$ are assumed to be 0.4 and 0.9, respectively [22].

## 3. The Proposed PSO–LWPLS Method

In this section, the bandwidth-tuning problem in LWPLS is first described. Then, why PSO was chosen is explained in detail. Finally, the proposed bandwidth optimization strategy is introduced.

### 3.1. Bandwidth Tuning Problem in LWPLS

**Figure 1:**
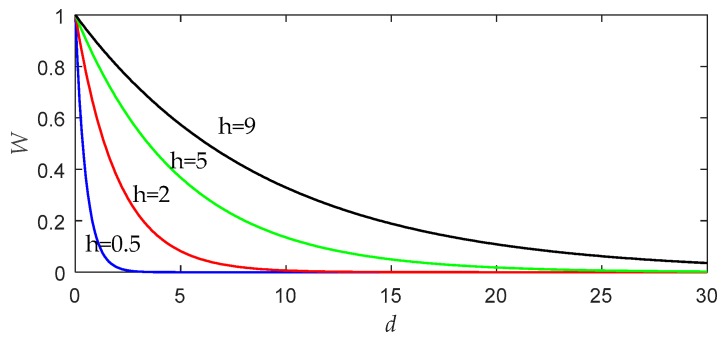
Variations of weight with distance under different bandwidth values.

Similar to most learning algorithms, the LWPLS algorithm also requires parameter tuning to achieve a good performance. In LWPLS, the bandwidth is an important parameter that defines the range of the neighborhood, and the shape of the weight changes when a local PLS model is constructed [20]. To illustrate the effect of bandwidth on the performance of LWPLS, we draw a graph, as shown in Figure 1, according to the weight function defined by Equation (3). 

As can be seen from Figure 1, the bandwidth *h* can control which samples play decisive roles in local modeling near the query point. If the bandwidth value increases, more remote samples will also have an opportunity to affect the query result. When *h* tends to be infinite, the similarity of all data becomes equal to 1, and the LWPLS algorithm will be no different from the simple PLS algorithm. On the other hand, the smaller the bandwidth is, the faster the weight decreases. In this case, only a few samples close to the query point are used for local modeling. If *h* is too small, the problem of overfitting will occur, and the reliability of the LWPLS model will also be affected [21,22].

Since the data density and distribution in the whole dataset are unlikely to be the same in different locations, it is necessary to design an optimal bandwidth for every query to ensure that every prediction can achieve satisfactory accuracy. In addition, online queries usually need to be answered in time, so computational overhead is also an important factor that cannot be ignored in bandwidth selection. Therefore, to improve the model’s accuracy without excessively increasing the computational overhead, a scheme is needed to determine the optimal bandwidth.

### 3.2. Why Use PSO?

To design an optimal bandwidth for each query is a single parameter optimization problem. Why is PSO used instead of one-dimensional search methods such as the golden section search method (GSSM) or the Newton method?

Based on the introduction of the LWPLS algorithm in Section 2.1, one can see that the bandwidth parameter initially appears in the calculation of sample weight in Equation (3). The weights undergo complex transformations in the LWPLS operation process, and they have been applied a number of times to calculate intermediate variables, such as in constructing the matrix $X_{r}^{T}\Omega Y_{r}Y_{r}^{T}\Omega X_{r}$ and solving eigenvectors to obtain intermediate variables, solving latent variables and load vectors in LWPLS, and so on. Therefore, after these complex calculations and transformations, one cannot exactly know the relationship between the prediction error and the bandwidth parameter, and cannot express the relationship in an accurate mathematical expression.

**Figure 2:**
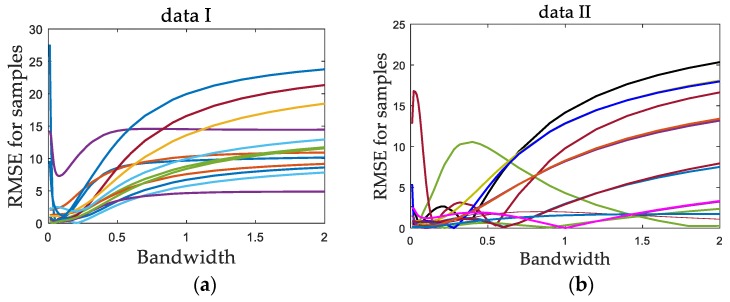
Root mean square error (RMSE) curves with different bandwidth values: (**a**) data set I; (**b**) data set II.

In addition, through numerical simulations, data set I with a uniform data density and data set II with a non-uniform data density are constructed. A series of bandwidth values are selected. For each data set, a number of sample points are randomly selected as test data. Each data set was tested independently, and the predicted values were obtained by using the LWPLS model. Then, for each test sample point, with each bandwidth value, repeated independent experiments were carried out, and the predicted root mean square error (RMSE) of the test sample was calculated. Then, the relationship curve of the RMSE with bandwidth values for each test sample was obtained, as shown in Figure 2.

It can be seen for data set I that each RMSE curve basically had a unique minimum value that changed with the bandwidth, which corresponds to the optimal bandwidth of the sample, and the optimal bandwidth values of all test samples were very concentrated. For data set II, because of the uneven distribution of data, the optimal bandwidth values of different samples were relatively scattered, and some RMSE curves had multiple minimum values.

The above two factors can be summarized as follows: first, the relationship between the objective function and the bandwidth parameter is complex and cannot be expressed by explicit formulas; second, there may be multiple minimum values in the objective function. These two factors make some common one-dimensional search methods, such as the golden section search method (GSSM) and the Newton method, not applicable. However, as a kind of swarm intelligence algorithm, PSO itself has fewer requirements for the mathematical nature of the optimization problem. Therefore, in this paper, PSO is used in the bandwidth optimization problem. To verify its effectiveness, the PSO method was compared with the popular golden section search method in Section 4.

### 3.3. Optimal Bandwidth Selection Using PSO

**Figure 3:**
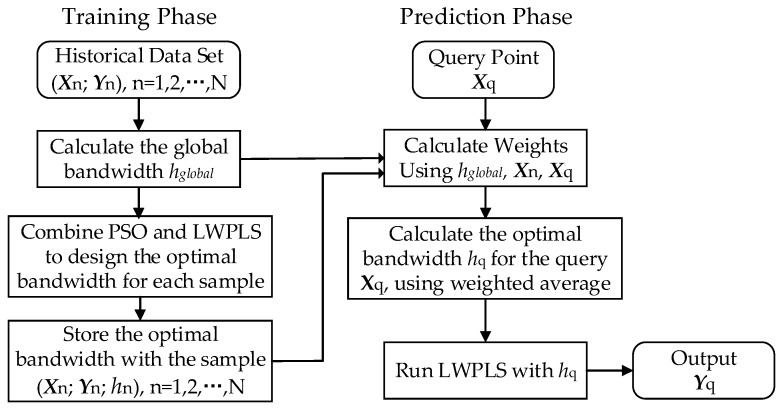
Framework of the proposed two-phase strategy.

In this section, we introduce the hybrid PSO–LWPLS with a two-phase strategy to meet the above requirements, including a training phase and a prediction phase. Figure 3 shows the framework of the proposed two-phase strategy. The idea of the point-based bandwidth selection and the K-NN method are combined together in this framework. The specific steps of the two phases are given below.

#### 3.3.1. Training Phase

In the training phase, an optimal bandwidth is designed for each sample in the historical data set, so as to prepare the parameters for the prediction phase. PSO and LWPLS are both used to calculate the optimal bandwidth. Before calculation, the initial parameters of the PSO algorithm need to be set. In order to determine the bandwidth selection range (*h*_min_, *h*_max_), we first adopted the leave-one-out (LOO) cross-validation method to obtain the best global bandwidth h_global_. The global optimal bandwidth represents the average of the optimal bandwidth of all samples, and the optimal bandwidth of each sample basically fluctuates near the average. In practical applications, one can first give a test range based on the global bandwidth, such as h_min_ = 0.5 h_global_ and h_max_ = 1.5 h_global_; then, the optimal bandwidth value of each training sample can be obtained by offline optimization calculations. If the optimal bandwidths of all samples are within this range, and the gap between the bandwidths and the range boundaries is not too large, it indicates that the above test range is reasonable. If the optimal bandwidths of some samples reach the boundary values, the range should be enlarged appropriately to ensure that each sample can search for the optimal bandwidth in the range. However, this range should not be too large to affect the convergence rate of PSO.

Although the whole training phase may seem time consuming, the computational cost is usually acceptable because all the calculations are done offline. The steps are as follows.

**Step 1**: Calculate the global optimal bandwidth *h*_global_ using leave-one-out cross-validation.

**Step 2**: Combine PSO and LWPLS to design the optimal bandwidth for each sample, as described in the following procedure.

For one sample (***X***_n_; ***Y***_n_) of the historical data set, when calculating its corresponding bandwidth, it is used as a query, and the rest are taken as training samples. In this optimization problem, the particles represent various bandwidth values in the range (e.g., *h*_min_ = 0.5 *h*_global_, *h*_max_ = 1.5 *h*_global_). The fitness function is defined as [21]:

(20) $$\mathrm{Fitness}=\frac{1}{1+{\left(Y_{n}-{\hat{Y}}_{n}\right)}^{2}}$$

where $\hat{Y}n$ is the estimated output by the LWPLS algorithm. Aiming at maximizing the fitness function, the optimal bandwidth *h*_n_ corresponding to the sample (***X***_n_, ***Y***_n_) can be finally obtained by iterations of the PSO algorithm.

**Step 3**: Store the optimal bandwidth *h*_n_ with the corresponding sample.

After completing the training phase, we can get a new data set with point-based optimal bandwidth, i.e., (***X***_n_, ***Y***_n_; *h*_n_), n = 1, 2, …, N.

#### 3.3.2. Prediction Phase

The task of the prediction phase is to design an optimal bandwidth *h*_q_ for the given query ***X***_q_ and estimate the output $\hat{Y}q$ according to *h*_q_. First, weights need to be calculated for every sample according to Equation (3). However, we note that before the optimal bandwidth *h*_q_ is obtained, the initial weight calculation itself requires a bandwidth h, which results in a Catch-22 situation [31]. To solve this problem, the global bandwidth obtained during the training phase is used to calculate the initial sample weights, because the global bandwidth is obtained by sufficient cross-validation on the training sample set, which also makes full use of the information provided by the sample data. Besides, there is no more information to prove which initial value is optimal. If the initial bandwidth is set at random, greater risk and error may occur. The detailed calculation steps are as follows.

**Step 1**: Calculate the initial sample weights $\omega_{hn}$ based on global bandwidth:

(21) $$\omega_{hn}=\exp(-\frac{dn}{hglobal\times \sigma_{d}})$$

where $\omega_{hn}$ represents the weight of the nth sample.

**Step 2**: Search through the entire training set for the K most similar samples and calculate *h*_q_ for the given query ***X***_q_, using the weighted average as follows:

(22) $$hq=\sum_{n=1}^{K}\omega_{hn}h_{n}/\sum_{n=1}^{K}\omega_{hn}$$

This is proposed according to reference [20], (p. 15), Equation (5), called “Weighting the Data Directly”. It is based on the principle that a similar input produces a similar output. Based on this principle, one can infer that the optimal bandwidth of the query point should be close to the optimal bandwidth of the neighboring points in the sample set, since the data density distribution around the neighboring points should be similar. In the training phase, the optimal bandwidth of each sample has been obtained, so to calculate the optimal bandwidth of the query point, one can first determine K neighboring sample points around it, and then calculate the weighted average of their optimal bandwidths—that is, the optimal bandwidth of the query point. The weighted average method makes *h*_q_ less sensitive to the change of K value, and the optimal value of K (basically between 2–10) is easier to determine by the trial-and-error method. When K continues to increase, the optimal bandwidth is basically stable due to the attenuation of the weights in the above Equation (22).

**Step 3**: Run the LWPLS algorithm with query-based bandwidth *h*_q_ to obtain the corresponding output estimation $\hat{Y}q$.

## 4. Case Studies

We discussed a numerical example and an industrial case in this section. Three common criteria, the root mean square error (RMSE), the mean absolute error (MAE), and the maximum absolute error (MAX), are adopted to estimate the performance of different methods. They are defined as follows:

(23) $$\mathrm{RMSE}=\sqrt{(\sum_{n=1}^{K}{\left(Y_{n}-{\overset{⌢}{Y}}_{n}\right)}^{2})/N}$$

(24) $$\mathrm{MAE}=(\sum_{n=1}^{K}|Y_{n}-{\overset{⌢}{Y}}_{n}|)/N$$

(25) $$\mathrm{MAX}=\mathrm{maximum}(\left|Y_{n}-{\overset{⌢}{Y}}_{n}\right|)$$

where $Y_{n}$ and ${\overset{⌢}{Y}}_{n}$ denote the real and predicted value of the *n*th test sample. *N* denotes the number of test samples. All the experimental calculations were performed on a computer with Intel(R) Core(TM) i7 -4790 CPU, Windows 7, MATLAB version R2015a.

### 4.1. Numerical Example

#### 4.1.1. Problem Settings

The first numerical simulation case used here comes from case 1 in reference [17] (p. 46), which was originally adopted to estimate the performance of LWPLS algorithms using different similarity calculation methods. This paper focuses on the effect of bandwidth optimization on the performance of LWPLS, so a modified numerical case, called case 2, is designed here. The two cases are described below.

Case 1

In this case, both input and response variables (***X***, ***Y***) are generated from three uniformly distributed, latent variables: ***S***_1_, ***S***_2_, and ***S***_3_. ***W***_0_, ***W***_1_, ***W***_2_, and ***W***_3_ are noise variables with a normal distribution. Here, we used rand(a, b) to represent the uniform distribution between two real numbers a and b, and we used N(μ, σ^2^) to represent the normal distribution with mean μ and standard deviation σ [17]. All variables are given below:

(26) $$W_{m}∼N(0,0.02^{2})(m=0,1,2,3)$$

(27) $$S_{m}∼\mathrm{rand}(-5,5)(m=1,2,3)$$

(28) $$X_{m}=S_{m}+W_{m}(m=1,2,3)$$

(29) $$Y=10S_{1}+5S_{2}{}^{2}+\exp(S_{3})+W_{0}$$

Case 2

This numerical case is a modification of case 1, in which uniformly distributed variables ***S***_1_, ***S***_2_ are changed to be normally distributed ones with a mean of 0 and standard deviation of 2, i.e., N~(0, 2^2^). The other variables remain the same as those in case 1.

**Figure 4:**
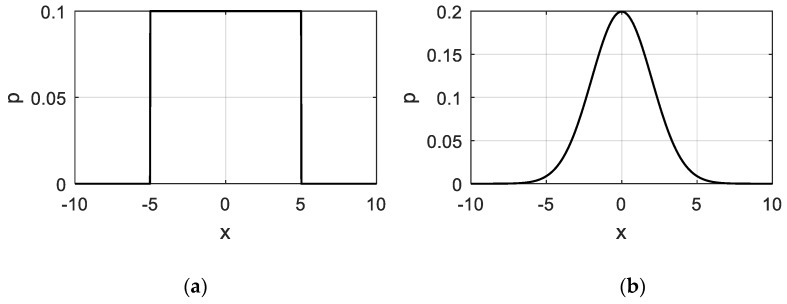
Probability density function: (**a**) rand(–5,5); (**b**) N~(0, 2^2^).

Figure 4a,b show the probability density distribution of the uniform distribution rand(–5, 5) and the normal distribution **N**(0, 2^2^). It can be seen that the data takes the values from –5 to 5 with equal probability in the uniform distribution, while in the normal distribution, most of the data concentrates near the mean zero, which results in distribution differences in different locations. Therefore, in case 1, since ***S***1, ***S***2, and ***S***3 are all uniformly distributed, the data density of the input space varies slightly at different locations; while in case 2, since ***S***1 and ***S***2 are normally distributed, the data density can vary greatly, although most of the data values are also in the interval [–5, 5].

For each of the above two cases, 500 samples were generated, 400 of which were taken as the training samples and 100 were taken as the testing ones. Four modeling methods—PLS, LWPLS, LWPLS with bandwidth optimized by the golden section search method, and PSO–LWPLS—were used and compared.

#### 4.1.2. Results and Discussion

In order to simplify the description, we first recorded LWPLS with the bandwidth optimized by GSSM as GSSM–LWPLS. Then, for the above two cases, PLS, LWPLS, and GSSM–LWPLS were used to build the prediction models and were compared with the PSO–LWPLS method proposed in this paper.

The prediction errors of the results for the two cases are shown in Table 1. The proposed PSO–LWPLS method had the smallest RMSE, MAE, and MAX in both cases. However, in case 1, the performance of PSO–LWPLS was not much better than that of LWPLS or GSSM–LWPLS. In case 2, PSO–LWPLS was considerably superior to the conventional methods. This is because the data density and distribution at different locations are almost the same in case 1, and the query-based optimal bandwidth is very close to the global one; the performance improvement was not significant. While in case 2, because of the influence of the normal distribution, the data density could vary greatly in the input space. In this situation, the fixed global bandwidth was not able to adapt to the density difference around the query points, resulting in large prediction errors. The bandwidth parameter was optimized in GSSM–LWPLS. Although GSSM usually performs well in the case of a single extremum, in the case of multiple extremums in the objective function, this method may not be able to obtain the global optimal bandwidth. The proposed PSO–LWPLS uses PSO to search for the global optimal bandwidth. PSO as a kind of meta-heuristics may also fall into local optimum, but it is more likely to find global optimum than GSSM, since particle swarms search a wider range than golden section optimization. Thus, the PSO–LWPLS method achieves much higher prediction accuracy, and it can greatly enhance the adaptability of the model to differences in data density.

**Figure 5:**
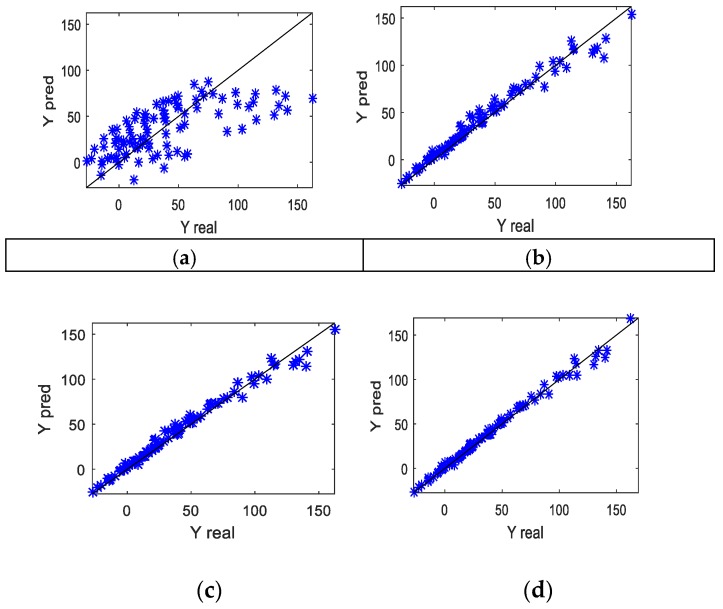
Comparison of scatter plots for the real and predicted Y values using four methods for case 2: (**a**) PLS; (**b**) LWPLS; (**c**) GSSM–LWPLS; and (**d**) PSO–LWPLS.

For the testing data set in case 2, scatter plots were used to illustrate the relationship between the real and predicted values, as shown in Figure 5. Figure 5a shows the results of the PLS method. It can be seen that the deviations between the real and predicted values were generally large. This is because the relationship between the input and output variables determined by Equation (29) was nonlinear. PLS is a linear modeling method, and cannot cope with nonlinear problems well. The results shown in Figure 5b are better than those in (a), which proves that the LWPLS modeling method has the ability to deal with nonlinearity, to a certain extent, by building a local model for every query, but its accuracy is still limited. Compared to Figure 5b, the performance of Figure 5c was further improved, but the improvement was not significant. Figure 5d shows an apparent improvement, which proves that the proposed method has obvious advantages over PLS, LWPLS, and GSSM–LWPLS in the case of an uneven density distribution. Since the distribution of actual industrial process data is usually complex and uneven, the proposed method is more suitable for practical application, and this will be verified by an industrial case below.

### 4.2. Application in the Production of Gray Cast Iron

#### 4.2.1. Problem Statement

**Figure 6:**
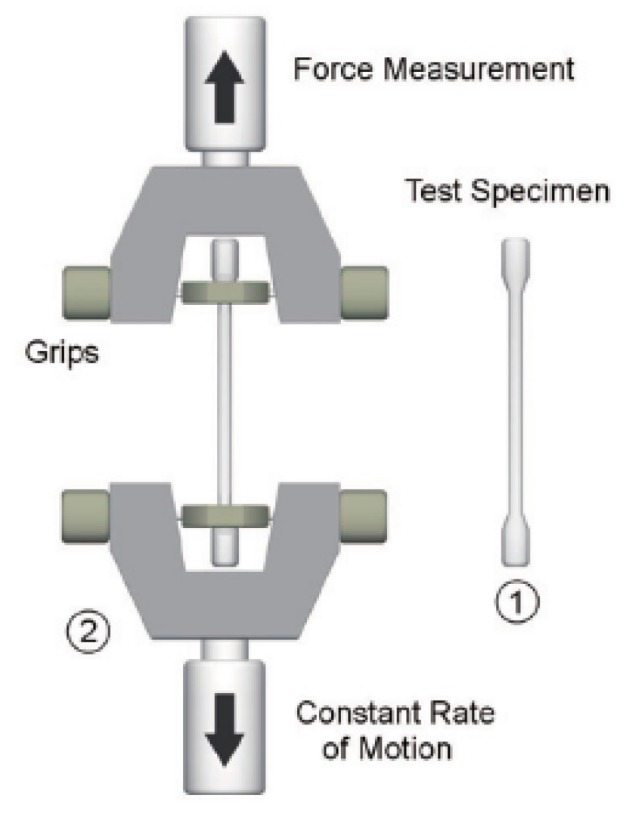
Tensile Strength test [32].

Gray cast iron accounts for a high proportion of all kinds of castings, and it has been widely used in many industrial fields. Tensile strength (TS) is an important mechanical property for gray cast iron, since it determines the scope of use and affects the service life of a cast component. At present, the tensile strength of a casting is usually measured by using a destructive testing method; that is, a testing specimen should be prepared at first, and then the tensile strength test is carried out, as shown in Figure 6 [32]. However, this testing method is very time consuming, and only a few samples in a batch can be tested.

In order to measure the tensile strength more quickly and easily, data-driven modeling methods have been introduced to predict the TS of gray cast iron, including multiple regression (MR) [33,34], back-propagation ANN (BP-ANN) [32,35], and so on. However, because of the chemical instability of raw materials or other changes in the casting process, the performances of these models could deteriorate, and even model failure may occur. Therefore, the above methods cannot be applied well to actual casting production. In this paper, we tried to use the proposed PSO–LWPLS hybrid modeling method to predict the TS of gray cast iron.

#### 4.2.2. Data Acquisition

The main chemical components (carbon, silicon, manganese, phosphorus, and sulfur) were selected as the input parameters and the tensile strength was selected as the output parameter. Among the components, carbon and silicon were measured by a thermal analyzer, and the rest were measured by a spectrometer. From 1 April 2018 to 30 October 2018, a total of 203 samples were collected from the production process of gray cast iron in a large foundry in Anhui Province. A total of 188 valid samples were retained after processing the abnormal values. Among them, 31 samples were randomly selected as the testing set, and the rest were taken as the training set.

#### 4.2.3. Multicollinearity Analysis

The existence of multicollinearity among variables will not only cause computational complexity, but also affect the accuracy and stability of the model [36,37]. In order to investigate whether there is a collinearity problem in the TS prediction of gray cast iron, the correlation coefficients between all input and output variables were studied first, as shown in Table 2.

From Table 2, it can be seen that the correlation coefficient between C content and TS was the largest, indicating that C greatly influenced the tensile strength. In addition, the correlation coefficient between C and another input variable Si reached 0.611. Therefore, we decided to diagnose multicollinearity by using the commonly used variance inflation factor (VIF), which is defined as:

(30) $${\mathrm{VIE}}_{i}=\frac{1}{1-R_{i}{}^{2}}$$

VIF*i* is the variance inflation factor corresponding to the input variable X*i* (*I* = 1, 2, …, 5); and $R_{i}{}^{2}$ is the determination coefficient, which is obtained by taking X*i* as the output and making regression on the remaining input variables. Based on Equation (30), the VIF values of five input variables C, Si, Mn, P, and S were obtained to be 25.4, 15.9, 1.2, 3.6, and 8.3, respectively. Since the maximum VIF value is greater than 10, it shows that there is multicollinearity among variables.

**Figure 7:**
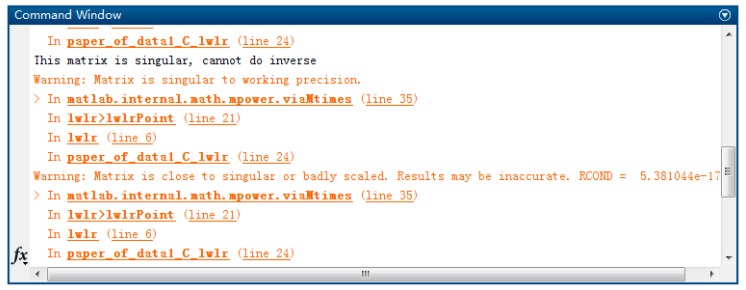
Matrix singular warnings during prediction using LWLR.

This is also reflected in our experiments. Initially, locally weighted linear regression (LWLR) was used for local modeling, which could not deal with the collinearity problem. In the test experiment, there were always some test samples that could not get exact predictive values, and MATLAB gave warnings, as shown in Figure 7 below. This also indicates that the collinearity problem may affect the stability of the model. Therefore, in the TS prediction of gray cast iron, we finally chose LWPLS, which can deal with the collinearity problem.

#### 4.2.4. Results and Discussion

The five methods, including PLS, BP-ANN, LWPLS, GSSM–LWPLS, and PSO–LWPLS, were adopted to build TS prediction models. The structure of BP-ANN was determined to be 5–7–1 by the trial-and-error method. Cross-validation was adopted to obtain an optimal global bandwidth for LWPLS. The number of latent variables of all PLS-based methods was set to be 4, according to the correlation coefficients between input and output variables. The relative root mean square error (RE) was also calculated here, as defined below:

(31) $$\mathrm{RE}=\sqrt{\sum_{n=1}^{N}\left[(Y_{n}-{\overset{⌢}{Y}}_{n}\right)/Y_{n}{]}^{2}/N}\times 100\%$$

The results of the TS prediction errors are shown in Table 3. The results show that the PLS method had the largest errors because of the unknown nonlinear relationship between the TS and input variables. Both BP-ANN and LWPLS have the ability to deal with nonlinearity, and the prediction errors are reduced to some extent. However, because of the possible variations in the casting process or the data density differences of training samples, the performance of these two models needed to be further improved. Compared with LWPLS with a fixed bandwidth, GSSM–LWPLS had a higher accuracy, which shows that GSSM has a certain effect on bandwidth optimization, but the improvement was not significant. The proposed PSO–LWPLS method achieved the best performance in dealing with the problems mentioned above.

**Figure 8:**
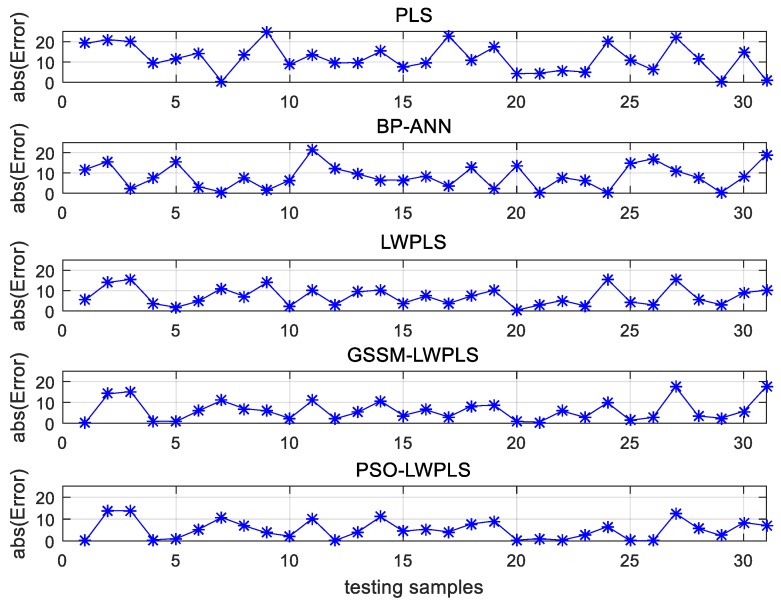
The predicted absolute errors for all the testing samples.

As shown in Figure 8, the absolute predicted errors of all test samples were also examined, so the performance of these five methods could be compared in more detail. It can be seen that the general absolute error trend of the proposed PSO–LWPLS method was the smallest among the five methods, which also means that the proposed method achieves the highest accuracy in predicting the tensile strength of gray iron.

### 4.3. Computation Time Analysis

In order to investigate the computational overhead, the computation times of the above five modeling methods in predicting the TS of gray cast iron were studied, including training time and prediction time (for answering all the testing points). The results are shown in Table 4. PLS and BP-ANN are global modeling methods, which are notably separated between the training and prediction phases. LWPLS, GSSM–LWPLS, and the proposed PSO–LWPLS are all JITL-based methods, which integrate the training and prediction phase into the entire calculation process when queries need to be answered. However, in this paper, the proposed method still contains a training phase when it is used for prediction, which is our innovative design to improve the prediction accuracy of the model. As shown in Table 4, although the training phase of the PSO–LWPLS method was relatively time consuming, the computation time of its prediction phase was not much longer than that of LWPLS. The computational overhead of the proposed method is completely acceptable in practical applications, and the improvement of its performance makes it absolutely superior.

Furthermore, in order to investigate the relationship between the computation time and the number of training samples, the changes of training and prediction times were studied by increasing the number of training samples in numerical case 2 in Section 4.1. The results are shown in Table 5. It can be seen that with the increase of training samples, the training time increased rapidly, but the prediction time (for one testing point) did not increase too much. This can also be explained as the increase of training samples made the training phase more time consuming, but it had little impact on the query response speed.

Actually, in our applied industrial case of gray cast iron, because the sample set itself was small in both training and prediction phases, we chose to involve all training samples when LWPLS was used to construct a model, and we let the bandwidth control the range of samples used and the contribution of different samples to the model. In other applications, if the sample size is large, in order to balance the computation time and the speed of training a model, we can select some neighbor samples from the sample set to form a small sample subset, and then we can build a local model on the subset with the proposed bandwidth optimization strategy. The selection of a neighbor sample subset is a common step in most just-in-time learning (JITL) methods, so we have not discussed this point in detail in this paper.

## 5. Conclusions

Locally weighted PLS (LWPLS) was used for industrial soft sensor modeling. To improve the performance of LWPLS without excessively increasing the computational overhead, a hybrid modeling method combining PSO and LWPLS was proposed, and a two-phase bandwidth optimization strategy was designed, including a training phase and a prediction phase. Every sample point in the historical data set obtained an optimal bandwidth by using PSO in the training phase. In the prediction phase, a weighted average method was designed to select an optimal bandwidth for each query, and then the prediction value was calculated according to this query-based optimal bandwidth. Through a numerical example and an industrial application case, it was proved that, compared to traditional global modeling methods and the LWPLS with a fixed bandwidth, the proposed method achieved a higher prediction accuracy, since it could better adapt to data density changes, and its online computational overhead did not increase too much. Next, we will try to further improve the computational efficiency of this method and explore its application in various industrial processes.

## Figures and Tables

**Table 1:** Statistical analysis of prediction errors. GSSM: golden section search method, LWPLS: locally weighted partial least squares, MAE: mean absolute error, MAX: maximum absolute error, PLS: partial least squares, PSO: particle swarm optimization.

Case	Method	RMSE	MAE	MAX
1	PLS	21.09	15.70	89.94
	LWPLS	4.20	2.59	15.47
	GSSM–LWPLS	4.09	2.52	15.20
	PSO–LWPLS	**4.07**	**2.49**	**15.15**
2	PLS	31.18	24.08	92.86
	LWPLS	7.58	5.77	31.28
	GSSM–LWPLS	6.74	4.91	25.63
	PSO–LWPLS	**4.02**	**2.81**	**15.66**

**Table 2:** Correlation coefficients between all input and output variables. TS: tensile strength.

Correlation Coefficients	C	Si	Mn	P	S	TS
**C**	1.000	**0.611**	–0.125	0.372	0.199	–0.738
**Si**		1.000	–0.156	0.298	0.302	–0.559
**Mn**			1.000	0.033	0.151	0.252
**P**				1.000	0.523	–0.351
**S**					1.000	–0.516
**TS**						1.000

**Table 3:** Statistical analysis of TS prediction errors. BP-ANN: back-propagation artificial neural network.

Method	RMSE	RE (%)	MAE	MAX
PLS	13.6	5.63	11.8	25.0
BP-ANN	10.8	4.38	8.9	22.5
LWPLS	8.5	3.44	7.2	15.7
GSSM–LWPLS	8.0	3.27	6.3	17.7
PSO–LWPLS	**6.8**	**2.84**	**5.3**	**13.9**

**Table 4:** Comparison of the computation times of the five methods.

Method	Training Time (s)	Prediction Time (s)
PLS	3.8	<1
BP-ANN	9.8	<1
LWPLS	3.7	
GSSM–LWPLS	5.1	3.9
PSO–LWPLS	100.7	3.9

**Table 5:** The computation time of PSO–LWPLS varies with the number of training samples.

Time (s)	Number of Training Samples						
	100	200	300	400	600	800	1000
Training time	33.6	98.4	212.6	498.1	1248.0	3749.2	10,046.7
Prediction time	<1	<1	1.0	1.0	1.1	1.2	1.4
